# M. Chomel on Acute and Chronic Amygdalitis

**Published:** 1842-06-04

**Authors:** 


M. Chomel *on Acute and Chronic Amygdalitis.*—A young man, 18 years of age, was admitted into the Hotel-Dieu with acute inflammation of the tonsils. He has been subject to this disease since the age of 11 years, having had thirty attacks since then. The inflammation was always confined to the left tonsil, and generally lasted from eight to ten days. The patient says that the tumour was opened only once, and that he never remembers any pus having been discharged from it. The first attack was the most severe, and with regard to this point M. Chomel directs attention to a principle which applies to all inflammations—viz. that when any organ is frequently the seat of inflammation, the first is always the most violent, and the subsequent inflammations decrease in intensity while they increase in duration. Hence, when any organ has been frequently the seat of inflammation it is extremely difficult to get rid of it, and it leaves a great tendency to to relapse under the slightest cause.

Inflammation of the tonsils may occasionally produce dangerous symptoms. In some cases the swelling of the gland may occasion imminent signs of suffocation. M. Chomel points out a mechanical means which he has often employed with success to relieve this state; he introduces the index finger into the mouth, and presses very firmly on the enlarged gland; a quantity of viscid fluid is thus discharged, and the tumour undergoes a momentary diminution of volume, which gives the patient great relief.

On examining the throat of the patient, now under treatment, the left tonsil and side of the velum palati are seen to be extremely swollen; and the mucous membrane of the latter part is raised up into the form of a tumour. All these parts are deeply injected, and of a purple red colour. On first looking at it, one would think that the mucous membrane of the palate was raised up by a collection of pus, but pressure with the finger shows that this is not the case; pus will, however, soon collect, but M. Chomel is not in the habit of opening the abscess with the knife unless under special circumstances.

The treatment of acute amygdalitis consists in general bleeding, emetics, and purgatives. They never, it is true, cut short the disease, but they moderate the violence of the inflammation. Of the three means, M. Chomel prefers the purgatives, as tending less than venesection to weaken the patient, and prevent him from following his ordinary occupations.

On the day of the patient's admission into hospital he was bled from the arm, a measure indicated by the fulness and hardness of his pulse; he then had an emetic, and was ordered to take some castor oil, and to use emollient gargles and the foot-bath. On the third day he was much better; he could swallow better, and the swelling at the back of the throat was much diminished. On the fifth day the inflammatory appearances all subsided, and on the seventh day the patient left the hospital well. The tonsil, however, remained affected with chronic enlargement.

M. Chomel delivered the following remarks on this case:—

The acute inflammation of the throat and tonsil, which, in the present instance, had supervened on the chronic, has completely disappeared under the influence of rest, and the treatment which has been employed. It is impossible to say whether suppuration had taken place or not; the patient spit up some whitish matter with his gargles, which had all the appearance of pus mixed with some streaks of blood, but void of that fœtid odour characteristic of pus discharged from the mucous membranes of the mouth and fauces. However this may be, the great tumefaction of the tonsil has subsided, and nothing now remains except the chronic swelling which always persists in cases of this kind. The patient may, therefore, be said to be cured of the acute disease, but the chronic one persists, together with the disposition to relapse under the least exciting cause.

We must then ask, by what means we can hope to prevent the return of the complaint? The use of borax, of alum, and other astringent substances has been proposed; but these means have so often failed, that surgeons have been compelled to have recourse to excision. We have often tried, in the Hotel-Dieu, scarification of the tonsil with considerable success; but this also failed in a great many other cases. Generally speaking, the chances of succes are less when the disease has been of long standing, and the tissue of the gland much hardened, and approaching the consistence of scirrhus. In cases like this, partial or total excision of the gland is the only measure that remains; but the operation should not be had recourse to lightly and inconsiderately, as has often occurred of late. I have seen patients who had been affected from childhood up, with enlargement of the tonsils, which had resisted every mode of treatment; excision was proposed as the only resource, and rejected; yet, after a considerable lapse of time, the disease has disappeared under the use of simple means, and probably, also, under the influence of time and the growth of the patient. When the latter, then, is young, it will be more prudent to abstain from any operation, and wait the effects of time.

Besides the species of inflammation now alluded to, there is another to which the attention of practitioners has been recently directed. This is an inflammatory affection of the posterior parietes of the pharynx, called *granular* angina. It is an inflammation of the same species as that observed in the mucous membrane of the stomach, and particularly in the neck of the uterus; an inflammation erroneously confounded with ulcerative or cancerous disease of that organ, to which it bears no relation. This form of angina is seldom met with in hospitals, because it is not sufficiently severe to compel working men to give up their daily occupations.

*Granular* angina is characterized by the following symptoms:—pain during swallowing or speaking; a sensation of tickling at the bottom of the throat; a constant desire to swallow or spit up the saliva; and uneasiness about the pharynx. On depressing strongly the base of the tongue, we perceive, at the back of the pharynx, a rough, irregular surface of a violet-red colour; this surface is covered with small spots or granulations, dispersed over various points, and covered with a matter resembling white of egg, or muco-purulent secretion. The granulations become gradually developed, and collect together so as to give the pharynx a mammellated appearance from which the disease has obtained the name of granular or mammellated angina.

The treatment of this affection is tedious, for, although not a severe one, it resists obstinately the remedies hitherto employed against it; in this respect resembling the granular disease of the uterus, a disease which often baffles the efforts of the physician. Besides, the affection of the pharynx is much more difficult to treat than the analogous one of the neck of the uterus. The latter organ is not very sensitive, and easily supports the action of very powerful topical applications, while the pharynx is so sensitive that the least touch causes excessive repugnance, and involuntary resistance on the part of the patient. Various means have been tried: in the first place, it was observed that this condition of the pharynx often coexists with various chronic affections of the skin, a remark equally applicable to the granular uterus; hence the use of alkaline and sulphureous baths was much insisted on; some benefit has been derived from these means, aided by purgatives and blisters; but the disease generally returns after having been cured for a certain time. The usual treatment of angina was then tried, and the nitrate of silver rubbed over the back of the pharynx; but the circumstances already alluded to render the application of these remedies difficult and inconvenient.

Hence I prefer the use of caustics in a liquid form, very concentrated, and past rapidly over the affected parts with a brush or pencil. This is, unquestionably, the best means that we can employ against a disease, one of the main features of which is the obstinate resistance that it offers to every species of treatment. In the use of caustics, however, we must be circumspect; we must proceed with the same caution that we employ in cases of granular disease of the neck and uterus—that is to say, not employ a painful and energetic mode of treatment at once, but gradually bring the patient to bear it, from one step to another.—*Prov. Med. and Surg. Journ.*, from *Gaz. des Hop.*, No. 28.

